# Miscellanies

**Published:** 1844-04-01

**Authors:** 


### Miscellanies.

### A Lecture on the Improvement of Medical Education in the United States. By M. Paine, M.D. &c. &c.

———————————— amphora cœpit
Institui—currente rotâ, cur urcens exit?—(Horace.)

The perusal of Dr. M. Paine's Lecture reminded us very forcibly of some passages of our old friend Horace; of the one, for instance, prefixed to this our notice, as well as of that wherein the poet describes the ludicrous effect which would be produced by the exhibition of a monster with a human head and a fish's tail. The lecture commences with a castigation of the chemical physiologists, for their presuming to encroach on the domain of physiology and pathology, and after raising our hopes, that the Doctor was about to enlighten us by grappling with and crushing the impudent pretensions and monstrous theories of Liebig, it abruptly breaks off into an entirely different subject, (an interesting one, no doubt), viz. that of the "Improvement of Medical Education," by which, however, we very soon found, to our utter amazement, the learned lecturer means neither more nor less than lowering the standard of professional "requirement," and that for no other reason, as he states in another part of the lecture, than because cheapness of education and a corresponding adaptation of time are found indispensable to the general condition of (American) society. Our respect for Dr. Paine induces us to take a regular, though necessarily a very cursory view of the more prominent parts of this lecture. Though we are little disposed to admit the validity of all Liebig's doctrines, or their adequacy to account for the various phenomena of disease, or to effect its removal, still our adhesion to the great principle of vitalism, shall never carry us so far as to make us utterly discard and repudiate the many valuable suggestions contained in "Chemistry applied to Physiology and Pathology."

We utterly deny that this work of Liebig's, recommended though it be, by its "insinuating simplicity," attempts to "promise an exemption from intellectual toil—to divest pathology and therapeutics, as well as physiology, of every intelligible principle—or to overthrow every other department in the Science." We suspect very strongly that the charge of "insinuating simplicity" had its real origin in the circumstance of Liebig's founding his theories, whether true or false, on plain matters of fact, and every-day observation, a mode of proceeding by no means congenial to the taste of those pathologists who have been all their lives mystifying themselves in the murky clouds of sublimated abstractions and fanciful hypotheses whereby to account for the various phenomena of health and disease.

With respect to Dr. Paine's learned coadjutor in the Quixotic attempt to demolish Liebig's theories, whose tirade, here referred to by Dr. Paine, with so much exultation, appeared, if we mistake not, in the North American Review, for October, 1842, we shall only say, that its vulgar verbosity, unbecoming personalities, and rancorous hatred of everything British, render it perfectly safe from our notice *non tali auxilio, nec defensoribus istis tempus eget*. Our confidence in the solidity of the foundations on which modern pathology is raised, is too great to allow us for a moment to apprehend that "the interference of the laboratory can ever shake the foundation or debase the science of medicine." At the same time we feel fully convinced that both physiology and pathology have derived, and will continue to derive, most valuable aids from the investigations and researches of modern chemistry. Having said thus much on the subject of Liebig's theories, we shall proceed to our notice of the other portion of the lecture. Dr. Paine says, " that from the necessity for medical practitioners in all assemblages of men, it is obvious, that provision should be made for the diffusion of medical knowledge, as far commensurate with the vastness and difficulties of the science, as the circumstance of each society will admit; and further, that there should be no limits to those requirements which humanity enjoins, and which education supplies, unless those imposed by the common exigencies of society." With this, as an abstract principle, we have no disposition to quarrel just now, at the same time that we cannot help expressing our horror of its practical application.

One of the main arguments advanced by Dr. Paine for requiring a low standard of medical education is, that this would tend to prevent the diffusion of quackery. Now, we beg leave to ask what advantages would result to society from sending out a set of imperfectly educated medical practitioners, for the purpose, forsooth, of preventing quacks from practising? Where is the difference between an ill-educated medical stripling furnished, it may be, with a few general principles, derived from lectures and from books, and a sheer quack, who has, at all events, scraped together a certain amount of empirical knowledge? Raise, says Dr. Paine, beyond a certain limited poise, our standard of absolute acquirements, and, I repeat it, we shall turn from our medical schools most of their aspirants into more humble channels, or into the walks of empiricism. The exigencies of American physicians demand an early application to the business of life. Now the plain English of all this is, require as small an amount of qualification as possible from the young medical aspirant—grant him a licence to practise without worrying the poor fellow's brain, or taxing his pocket too severely,—or the consequence will be—what? why, a set of men who never attended a single medical lecture—whose knowledge of disease is purely empirical, will step in and seize the practice—and a good job we think it would be for the public. " If we would cultivate the field of medicine," says the Dr. " we must look for an early harvest, or, my word for it, it will be soon over-run with weeds." We shall merely say, in reply to this, that if unqualified practitioners are let loose to cultivate the field of medicine, this field will be soon over-run, not " with weeds," but with the bodies of their unfortunate patients. Oh! but then, says Dr. Paine, when the harvest begins, then is the time for the most salutary stimulus of ambition;—that is, in other words, grant these poor fellows the licence to practise physic—to reap the harvest (and without sowing too), to treat disease (without understanding a tittle about it)—and then see what industrious students, what successful practitioners, they will become after a while—on the principle no doubt—*possunt quia posse videntur.* Surely, the uneducated quack can do all this, as well as the licensed practitioner, and so there is scarcely a difference—*mutato nomine,* &c.

One or two words more, and we have done. Dr. Paine says, " it is no small part of our surrounding advantages, which go to the formation of character, of judgment, and practical habits, at the very outset of professional life that our pecuniary wants turn us, almost from the cradle, to thoughtful habits of economy, and into the ways of industry. We thus early become familiar with man in all his aspects;—and with characters formed for decision, by participating in the events of society, and early accustomed to mark the varieties of the moral constitution, we are better qualified than they, who spring from the nursery of wealth, to appreciate the various conditions of the physical, and to carry our habits of business into the chamber of the sick." This is a truly graphic description of the great blessings of poverty. What a school for pathological and therapeutical attainments our American friends possess! What a splendid preparation for forming decision in medical treatment is afforded by the fact of the American medical student being put to his shifts, from his very cradle, in order to keep body and soul together! When a man is on the brink of starvation from want of means to procure a meal, he certainly needs decision. We cannot, however, for the life of us, suppose that it is the same kind of decision as that required in treating a difficult case of disease. To form a decision in the former case, mere instinct will almost suffice—but to form a decision in the latter case, considerable mental cultivation—experience acquired by well-directed attention to the various branches of medical science—a close and attentive observation of disease in hospital practice under the eye of an experienced and well-educated practitioner—these, and all these, are indispensably necessary, and for these the poverty of poor Job himself, can afford no adequate substitute. Poverty may give cunning—shrewdness—and habits of economising pecuniary resources—it never can impart tact in the diagnosis, prognosis, or treatment of disease.

---

### MEDICAL SCHOOLS IN ENGLAND AND AMERICA.

For the last two or three years the English, and, more particularly the London Schools of Medicine, have been declining in point of numbers. Several causes have operated in occasioning their falling off in the metropolitan establishments, but probably the chief one has been the recognition of Provincial Schools, and their consequent extension.

Whether this will ultimately tell for the benefit of medical education in Great Britain, we will not stop to inquire, although we are disposed to doubt it. When classes fail to remunerate lecturers, they will probably not receive that amount of attention and zeal inspired by the expectation of a liberal reward, nor will men exclusively engaged in practice devote their time and talents to an unprofitable drudgery. The consequence will be, that the student will lose the benefits derived from the experience of his instructor, benefits which it is impossible to over-estimate. If, indeed, the Provincial Schools were generally thriving, one would be disposed to overlook the damage done to the London ones, and, on the Benthamite principle, of the greatest good to the greatest number, strike the balance in favour of the change. But we are mistaken if the Provincial Schools *are* thriving. They abstract enough from the London Schools to injure *them*, but not enough, materially to benefit themselves.

It would seem to be otherwise in America. In the Medical Examiner,* we find a statement, which shews that the Schools throughout the United States are highly prosperous this year.

"Information from all parts of the country shows an unusually large attendance of Students at the various Medical Colleges, the present Winter. This may be accounted for by the embarrassed condition of agriculture and commerce during the last few years, which has probably induced more than the usual proportion of young men to look to the learned professions; and partly, perhaps, by the fact that many have been kept back by the general pecuniary distress which has prevailed, who now find means to avail themselves of the advantages of public lectures. At the two Schools in Philadelphia, the number of Students is greater than at any former season. The University of Pennsylvania has more than her average class, and the number at the Jefferson Medical College exceeds that of last year, by nearly one hundred."

In this country, one cause which has been assigned, and apparently with reason, for the low state of the classes, has been the depression of the manufacturers and agriculturists. If they can barely manage to get on, they are not in a condition to give their sons an expensive education. Add to this, the diffi-

* Jan. 13, 1844.

culty of providing for them afterwards, in consequence of the crowded state of the profession, and part, at all events, of the present state of things is accounted for. In America it seems to be just the reverse. The embarrassments of the farming and trading interests are assigned as causes of the prosperity of the schools. This would seem to shew that medical education is cheap, and medical remuneration in the New Country sufficient to obtain for the neophyte a living.

We cannot but remark with satisfaction the fortunate condition of the University of *Pennsylvania*. The "drab-coloured men" may well make doctors of their sons, seeing that they can pay the matriculation fee with other people's money. We only trust that that very fee is not *repudiated*, and that in that very respectable State, there is in their dealings with each other, honour among thieves.

---

### Loss of the Eye-brows and Eye-lashes from (supposed) Parasitic Animalcules.

Without going into the details of his discovery (if discovery it be), we may observe, that Mr. G. Robinson procured from a lady, who had lost her eyebrows and eyelashes an insect and some ova, which he believes were the cause of the loss in question.

The complaint was first observed about ten years previously, viz. in the autumn of 1832 or 1833, when the eyebrows gradually became so much detached from their connexion with the skin that, each time the face was washed, some half-dozen adhered to the towel. An intense itching of the skin covered by the eyebrows was then also, for the first time, remarked. This sensation became almost intolerable on entering a warm apartment, or on applying to the part any warm or stimulating substance. This irritability of the skin, doubtless occasionally aggravated by instinctive attempts to relieve it by friction, caused the integuments of the eyebrows constantly to assume a fiery red colour. Various applications were ineffectually employed, and, at length, after enduring the annoyance of the complaint during a period of four years, and being tormented during the whole of that time by numberless local applications, the chief effect of which was a momentary increase in the irritability of the skin, the morbid sensation and the loss of hair gradually ceased, and have never since returned.

Supposing insects to be the cause of her disorder, she preserved all the scurf, &c. from the affected part, and gave it to Mr. Robinson for examination. This was in 1842.

"On examining the whole of these particles under the microscope, I found, as might be expected, the greater part to consist of minute portions of epidermis, the scales of which were matted together, and slightly stained by a bloody serum. But with these there were also distinctly visible two bodies, which, from their shape, and from the firmness with which they preserved it under pressure, were evidently the ova of some small insect. In one of them the process of development had already commenced; for on it were seen six minute protuberances, the relative position of which indicated them as rudimentary legs.

"The larger and darker object, though slightly mutilated in consequence of the great length of time during which it had been preserved, was nevertheless sufficiently perfect to enable its nature to be satisfactorily ascertained; and it presented all the characteristics of a fully developed insect. Its body, oval in form, was much wider posteriorly than anteriorly; the legs, six in number, were chiefly remarkable for being long, flexible, and tapering. It should be remarked that this was the only perceptible insect that was ever obtained from the eyebrows; though the minute oval bodies were not unfrequently met with."

He was unable to obtain any accurate information on the nature of the insect, when he accidentally discovered the same on the body of the common house-fly. He has since found flies to be extensively infested with them. He believes that the complaint is occasioned by them, and suggests that the best preventive measure, in addition to general cleanliness, would be afforded by regularly brushing the eyebrows, night and morning, during the summer months. For the full-grown insect is sufficiently large to be removed by a brush; and if any had lodged there during the preceding interval, they might in this manner, be swept away before depositing their ova in the skin. And that the fully-developed insects can be thus displaced is rendered probable by the fact, that during the four years the complaint existed in the case here detailed, only one full-grown specimen was met with.

As to the treatment, he leaves that open, contenting himself with stating that it is not very easy, and finishing with two questions:—

1. " To what extent are the parasitical insects affecting the exterior of the human body, that of scabies for example, identical with, and derived from, those infesting the bodies of certain of the lower animals?

" And secondly, are not several, if not all the forms of pruritus—complaints of the incurability of which I have more than once observed, in the pages of this and other medical journals—occasioned by the lodgement of some minute parasitical insects in the skin, and not by mere inanimate dirt?"

Very possibly, but it must be owned that Mr. Robinson has not quite proved that the insects were the *cause* of the disorder, and not accidental complications of it.

---

### MEDICAL REMUNERATION IN EMIGRANT VESSELS.

The profession is put to hard shifts. Its numbers have cut down, and are still continuing to cut down, the remuneration of individuals to starvation-point. We all of us recollect the advertisement that appeared a little while ago, for the *place* of medical attendant on an invalid, there being no objection to undertake, at the same time, the duties of a valet. Such a gentleman, should he obtain the object of his ambition, would be ten times better off than the surgeon of an Union, or, as it appears, of an emigrant-ship. Mr. Churchill communicates to the *Lancet* from the Colonial Magazine, a calculation of the pay and profits of the latter hazardous employment. Here it is:—

| | | |
|---|---|---|
| 200 emigrants (and that, if anything, exceeds the average number, although some few vessels have taken 300 and upwards) at 10s. 6d. . . . . . . . . . . . . . . . . . . . . . . | | £105 |
| Expenses in harbour for six weeks (and where there are ineligible emigrants full that time will be expended in investigating whether the surgeon was ignorant of their being so on embarking), in correspondence, &c.; and an average of 2*l.* per week all who have visited the colony can attest will not be more, in the towns, than sufficient to supply actual necessaries . . . . . . . . | £12 | |
| Additional outfit for the voyages, out and home, to the apparel that may be considered requisite if otherwise employed, at but the small sum of . . . . . . . . . . . . . . . . . | 20 | |
| And the homeward passage money, to do duty as surgeon, at the average (which, if anything, is under the mark) of 40*l.*; and saying such is obtainable in the period of six weeks, what is the balance of the bounty, after the hazard and anxiety attending such an undertaking . . . . . . . . . . . . . . . . . | 40 | 72 |
| Balance . . . . . . . . . . . . . . . . . . | | £33 ! |

Not nearly so good as a man-servant's wages in a decent family.—*Lancet*, Feb. 24, 1844.

---

### Leeches good Weather-Guages.

" It was interesting to notice the leeches which I brought with me from Sydney—their movements in the bottle always denoting a change of temperature and weather; they always crawled to the top of the bottle on the approach of mild and agreeable weather; but were as sure to drop down and lie meshed together in an inactive and dormant state at the approach, and during the continuance of stormy and cold weather. They showed indications of a change from eighteen to twenty-four hours before we could ourselves anticipate any. I found a little sugar added to the water a great improvement. The water need only be changed once a fortnight in cold regions, but oftener in warm or tropical climates, varying the quantity of water to the number and previous applications of your leeches: a little fine sand is very desirable, and seems to be liked by the leeches, and with it they are less apt to prey upon each other."

So writes Dr. Thompson, who had charge of a convict-ship.—*Medical Gazette*. Jan. 26, 1844.

---

### Apothecaries' Diploma in Paris.

If we may trust the *Lancet*, the Diploma of Apothecaries's Hall is held cheap in Paris. We read that:—

" A Mr. Edmonds, practising in Paris, was lately summoned before the police court of the department, for exercising medicine in France without having received a licence from one of the faculties of medicine in that kingdom. The authority to practise possessed and laid before the court by Mr. Edmonds, was the certificate of the Master, Wardens, and Worshipful Society of Apothecaries. The president remarked that 'this diploma seemed to give authority to practise pharmacy only, and not medicine;' to which the practitioner replied, by urging that 'doctors, so called, in England, were required only in consultation, while apothecaries, otherwise surgeons—in fact, 'general practitioners'—were the medical attendants of the great mass of the population, furnishing medicines, and answering in some degree to the French *officiers de santé*, and that it was by virtue of the very kind of document which he produced that the general practitioners exercised their functions.' But this sage explanation failed to make clear to the president by what reason the licence of a company which he considered was trading in drugs should give the right to meddle with life and limb, and Mr. Edmonds was fined five shillings and costs. No account is given however, of his having attended medically any but his own countrymen residing in France."—*Lancet*, Sept. 30, 1843.

It certainly does seem hard that the English abroad may not be physick'd after their own fashion. If a German, in London, chooses to live or die by hydropathy, or Hahemannism, or any other mysticism of his country, nobody says nay. The Legislature acts on the principle:—

" What's that to me, I let's em."

And if a Frenchman in England chooses to be glyster'd when we think he should be blistered, he is still permitted, nemine obstante, to take his lavement or his demi-lavement undisturbed. Surely, then, it is particularly hard upon John Bull, to deprive him of his apothecary, and force him to take to a physician. Non tali auxilio, &c. say we.

---

SIMPSON'S NEW INSTRUMENT FOR CRUSHING CALCULI IN THE BLADDER. Shewn at the Royal Medico-Chirurgical Society, on Tuesday, March 12th, 1844.

Mr. Simpson, the ingenious surgical instrument maker, in the Strand, has addressed this letter to us, and shewn us the instrument referred to: it seems to us likely to be very serviceable in the cases for which it has been constructed.

Having been applied to a few weeks back in a case where the stone was supposed to be too large to extract without first breaking it, and as the instruments that have been hitherto made, have proved useless, in consequence of the difficulty, or rather impossibility of opening the blades of the Forceps after their introduction into the bladder, so as to grasp and crush the stone, partly from the great thickness of the blades, and partly from their being fixed together, I have constructed the instrument described in the following lines, from which I consider it will be obvious, that as the blades are introduced separately, and the stone may be comparatively easily crushed, the important and so long desired object is at length attained, namely, of being enabled to break up a large stone, and extract it by fragments, without greater danger than that usually attending the ordinary operation of lithotomy.

This instrument which is for the purpose of crushing calculi that are found to be too large to extract by the ordinary operation of lithotomy; consists of two strong, curved, flattish blades, rather more than three inches in length, which, together with the handles, makes the whole length of the instrument about fourteen inches. The blades are introduced into the bladder separately, so as to get round a large stone more easily. After the stone is seized between the blades, they are locked or connected together by means of a button joint, something similar to that of Rigby's Midwifery Forceps. They then resemble a pair of very strong, large-sized Lithotomy Forceps. After the blades are locked together, a flattish bar with a small screw cut on the edges, is fixed by means of a screw to one side of the Forceps, and passed through an opening made for it on the other. On this screw and outside the handles of the Forceps, a washer is first placed, and then the handle with the female screw is put on the bar, and by turning it on the screw bar, the handles and blades are gradually closed together. Should the stone not be very hard, this power may be sufficient to crush it, but if not, a slide fits into an opening in the screw bar that serves to close the blades. This slide fixes by means of a screw, in the centre, according to the width the blades may be opened, and a drill is passed through the hole in the slide, (in which a screw is cut for the purpose), and also through a swivel at the lower end of the Forceps, almost under the joint. A blunt Gorget may be passed into the bladder to guide the drill, and prevent its touching any part of the wound. The handle of the drill is then turned round and round, till it arrives at a stop placed on the drill to prevent its passing beyond the ends of the blades of the Forceps, and injuring the bladder, thus boring away the centre of the stone, and consequently considerably weakening it. The blades may then be closed by turning the handle on the screw bar, and thus crush the stone to pieces. Should the first hole not weaken the stone sufficiently, the Forceps can be opened, and the stone loosed from their grasp, and by moving the stone, and seizing it in a different position, bore another hole, but the probability is, that in most cases, the one hole would be found quite sufficient. The stone having been broken into small pieces, they can then be extracted by the usual Forceps or Scoop in the ordinary way. The length of the incision in the bladder required to introduce and use these Forceps, is not more than that usually made for the ordinary operation in the average of lithotomy cases.

There is also another pair of Forceps of about the same length, but with straight blades, and made much stronger, to be used in the same manner as those already described, but so as to enable the operator to crush the stone, without having recourse to the drill at all.

### Death from Hydropathic Treatment.

It has always seemed to us that ingurgitating the quantity of water which hydropathic quacks prescribe and hydropathic blockheads swallow, must tend to excite the kidneys overmuch, if not to produce disease in them. The following case is in point. We are not sanguine enough to suppose that it will go far towards curing the public of its mania for hydropathy. That is founded on too ingrained a love of quackery and of the marvellous, on too essential a constituent of the human mind—credulity— to be likely to be removed by many such cases as the following :—

Mr. C., aged 52, a druggist, in Nottingham,having only slight head-ache, was induced to take take to hydropathy. Previously to the commencement of this Mr. C. had been accustomed to take one glass of ale at dinner and one at supper, with an occasional glass of wine once or twice a week, but was a man of great regularity, very domestic and of spare frame. During the last days of August he commenced the cold water plan of treatment (discontinuing all fermented drinks,) by swallowing, during the day, from seven to eight pints of cold water, sponging night and morning, and taking a considerable amount of exercise. This system was continued until the commencement of December, when Mr. C. began to find himself becoming thinner and weaker ; that the quantity of urine passed was disproportionately great to even the large quantity of water taken ; that the tongue became red and dry, the thirst excessive, attended with a dry state of skin. From this time it is impossible to ascertain the quantity of water drunk, as Mr. C.'s thirst induced him to take it in greater quantities and drink more frequently than he had previously done.

This state of affairs continued until Thursday, the 21st of December, when he consulted Mr. Hawkesley, who found him with these symptoms :—Exhaustion very great; much emaciation ; pulse small, feeble, and fluttering, beating seventy-two in the minute ; skin cold and dry ; the thirst and appetite very great ; lower extremities cold ; urine in great quantity, he having passed for some time from five to six quarts during the twenty-four hours, limpid, insipid to the taste, reaction on test-paper acid, specific gravity 10.25. Notwithstanding the frequent exhibition of stimulants, these symptoms continued with little or no improvement until the Wednesday following, when the stomach became very irritable, and vomiting commenced, solids as well as liquids being rejected. Dr. Hutchinson now visited him. An opiate was prescribed; effervescing medicine, with hydrocyanic acid, and the tincture of sesquichloride of iron, to be taken as frequently as the state of the stomach would permit; beef-tea, brandy, with soda-water, and small portions of solid food. Mr. C. passed a good night and appeared better during the next day; but towards the evening the vomiting returned with increased frequency, and continued during the greater part of the night, until completely relieved by the frequent administration of one drop of creosote. Nevertheless, the exhaustion increased although stimuli were used very freely, both internally and externally; these had the effect of rousing him for a brief period, but every effort to rally him for any continued time proved unsuccessful; he gradually sank, and died at half-past one o'clock, p.m., on Friday.

The principal appearances disclosed on dissection were œdematous lungs—an anæmiated condition of the viscera—softening of the intestinal mucous membrane—deep-coloured softened kidneys, with small bloody points on section.*

No reasonable man, possessed of very moderate medical information, can doubt that hydropathy was the cause of death in this instance The hydropathists, of course, ready, like all impostors, to swear black is white, have the impudence to lay the patient's death on the hydrocyanic acid, &c., taken to allay

---

* *Lancet*, Jan. 13, 1844.

vomiting. The old story. When Morrison purged people to death with his pills, he protested that they died because they didn't take enough: and so it is with the water doctors. They drench their victims into diabetes or diarrhœa, and when they sink, it is either because they did not drink sufficient, or because what they did drink was drunk unskilfully. As if there was any skill or judgment in telling men to swill cold water all day long, and macerate themselves in it, as if they were to be made an infusion of.

By the way, the case of the late Sir Francis Burdett is another precious specimen of "the skill and judgment" of hydropathic practitioners. A man subject to gout, of an advanced age, is made to sit in cold water, imbibe enormous quantities of it, and totally change all his habits of life. What would any rational creature expect? Just what happened—congestion of some internal organ and the worst consequences. We were told about the time that Sir Francis began his hydropathic practices, of what the poor man was about. We predicted to our informant that he would live about a month, if he persisted in his folly. He died at the expiration of three weeks.

We are not such fools as to argue this matter with the public, nor such children as to be angry with the said public about it. People have, we suppose, a right to quack themselves if they choose. And if they *will* believe every knave who professes himself possessed of a grand secret for curing diseases, there is no remedy for it but their own experience of his roguery and their simplicity. As for putting down credulity by Act of Parliament, the thing is utterly preposterous. The law-makers themselves are the weakest dupes of any.

---

### BENZOIN WATER.

The sparing solubility of the salts of lithic acid is well known to be the proximate cause of a large proportion of calculous deposits.

It has been shewn that when benzoic acid, or one of its alcaline salts, is introduced into the stomach, the lithic acid of the urine becomes converted into hippuric acid, whose alcaline compounds are of very easy solubility, thus—

*Hippurate of Soda* is soluble in two parts of water, at 60° Faht., whilst

*Lithate of Soda* (the staple of gouty concretions, or "chalk stones") is not soluble in less than 4000 parts of water, at the same temperature.

Biborate of soda is possessed of a considerable disintegrating power over urinary calculi.

The solvent properties of the alcaline carbonates is strongly illustrated by the effects of the alcaline waters of Vichy, in dispersing the chalk stones of gout.

It is anticipated that by combining the several medicinal agents alluded to the efficacy of each will be enhanced, and that, whilst an excess of carbonic acid gas will gratify the palate and invigorate the stomach, copious dilution will hasten the assimilation of the remedies, and contribute towards their final operation. Accordingly each of the bottles hereby submitted to your consideration, contains, of

| | |
|---|---|
| PURIFIED Benzoate of Potash<br>Biborate of Soda - - - | of each 15 grains. |
| Bicarbonate of Potash - - | half a drachm. |
| Distilled Water - - - | 16 fluid ounces. |

which solution was prepared under a pressure of 2½ atmospheres of carbonic acid gas. The water will be found to retain a large proportion of its gas long after exposure to the air.

This "*Benzoin Water*" will be found to unite the properties of an antacid, of a diuretic, and of a tonic. It will be found useful in an irritable state of the mucous membranes, whether manifesting itself in dyspepsia, or in chronic bronchitis; in all cases where there is a disposition to the formation of earthy deposits, in whatever part of the human frame—and particularly where such formations are the result of an excessive generation of lithic acid in the system. —(Gout and Lithiasis.)

---

### Charter of the Royal College of Veterinary Surgeons.

Our friends need no longer call themselves *Veterinarians*. They are now a profession, and have all the trappings of ourselves. Victoria has done this for them sponte suâ. The *Times* informs us:—

Her most gracious Majesty the Queen has been pleased to grant to Thomas Turner, W. J. Goodwin, Thomas Mayer, William Jewell, William Dick, Charles Spooner, and James Beart Simonds, and those who now hold certificates of qualification from the Veterinary College of London, or the Veterinary College of Edinburgh, a Royal Charter of Incorporation, that they may be henceforth one body politic and corporate, by the name and title of the Royal College of Veterinary Surgeons. By this charter the veterinary art is recognised as a profession, and the members of this college are solely and exclusively of all other persons deemed members of the same, and distinguished by the name of Veterinary Surgeons. The regulation of the affairs of the college is vested in a council consisting of a president, six vice-presidents, and twenty-four members, all of whom are to be elected by the members at large. The council have the power of making by-laws, &c. and electing examiners to examine those who now are, or hereafter may become, students of the Royal Veterinary College of London, or the Veterinary College of Edinburgh.

---

### Observations on the Trees and Shrubs proper for our proposed National Cemeteries. By R. Dickson, M.D.

Dr. Dickson's object in this paper is to point out the kind of trees and shrubs which should be excluded from cemeteries. Those trees which are known to diminish, instead of increase the amount of oxygen, are no fit portion of the vegetation of cemeteries; strange to say, however, these have been held to be, almost by prescriptive right, the appropriate occupants of the spots set apart for the dead. Evergreens generally are allowable, but all containing volatile oils, or volatile constituents, which change into resin by the absorption of oxygen, are very unfit.

After excluding the coniferous plants above enumerated, there will still be enough of perennial leaved plants left to maintain an appearance of freshness and life throughout winter, the cherry-laurel (cerasus lauro-cerasus), the Portugal laurel (cerasus lusitanica), holly, ilex aquifolium, and above all, the arbutus unedo, and arbutus andrachne. Where walls surround the cemetery, the mespilus pyracantha, and other shrubs might be advantageously trained against them. Among deciduous plants, the poplars, especially the populus balsamifera, should be excluded. Along the sides of large cemeteries the ash might be planted, as the ornus Europœa, or flowering ash. One of the most appropriate would be the common lilac (syringa vulgaris), the leaves of which, according to Dr. Daubeny, raise the proportion of oxygen, in a jar filled with common air, to 29 or 30 per cent, and by introducing several plants into the same jar in pretty quick succession, even raise the proportion from 21 (the ordinary amount) to 39 per cent. In conformity with this, Dr. Dickson would exclude the cypress, yew, cedar, and arbor vitæ. One result of their banishment would be, that a more cheerful aspect would appertain to the spots where the remains of our departed friends repose.—*Provincial Medical Journal,* March 2, 1844.

---

### SIR HENRY HALFORD.

At the mature age of 78, and capable of exercising his profession, with vigour, till a year ago, died Sir Henry Halford, the most eminent Physician in England—if the most extensive practice among the highest ranks in life, be any criterion of eminence. Contemporary with Baillie, the two stars of the medical horizon shone with nearly equal lustre; but with different kinds of light. Dr. Baillie was sought for his real knowledge of MORBID ANATOMY, and his supposed knowledge of pathology—two things as different as cause and effect. His great competitor preferred no claim to deep science in either anatomy, physiology, or pathology; but he had a quick apprehension, an elegant address, a ready explanation for every phenomenon, a facile prescription for every symptom—in one word, he had TACT, of a very superior kind, which counterbalanced, and more than counterbalanced, all the drudgery of the dead-house, through which Dr. Baillie had waded.

Then again, his polished manners, his classical education, and his double-refined powers of flattery, gave him infinite advantage over his plain, homely, and unpolished rival, among the Royalty and the Aristocracy of the Kingdom, male and female. Dr. Baillie was summoned in dangerous cases, through fear, rather than favour; but Sir Henry was generally there long before the disease came to that pass, and consequently had the cream of the jest, while the pathologist had merely the honour of taking precedence of the undertaker.

Sir Henry's career lasted half a century, while that of Baillie ran only about 25 years. His figure was slight and elastic, his countenance animated and cheering, his activity of mind and body remarkable, and he had the art of inspiring hope in the breast of the expiring sufferer.

It was said that he prescribed chiefly for *symptoms;* but we know so little of the essential nature of diseases, that nine-tenths of our practice are, or ought to be, on this principle. How do we recognize maladies but by their symptoms; and have we any right to despise them in the treatment? It is to the *symptoms* of a complaint that the patient constantly refers—it is from these that he suffers—it is for them that he solicits the aid of the physician. It is therefore the duty as well as the interest of the physician to endeavour to alleviate symptoms, whatever else he may do in speculation on the nature and seat of the malady.

It appears that Sir Henry died of one of those affections on which he had written—NEURALGIA. Of late years, his advanced age, his separation from Court, the death of most of his old patients, and the pressure of a redundant doctorate, curtailed greatly the range of his practice; but it is probable that he realized more money by his profession than did any physician since the days of Hippocrates.

Quiescat in pace!

